# *In Vivo* Zonal Variation and Liver Cell-Type Specific NF-κB Localization after Chronic Adaptation to Ethanol and following Partial Hepatectomy

**DOI:** 10.1371/journal.pone.0140236

**Published:** 2015-10-09

**Authors:** Harshavardhan Nilakantan, Lakshmi Kuttippurathu, Austin Parrish, Jan B. Hoek, Rajanikanth Vadigepalli

**Affiliations:** 1 Daniel Baugh Institute for Functional Genomics and Computational Biology, Department of Pathology, Anatomy and Cell Biology, Thomas Jefferson University, Philadelphia, Pennsylvania, United States of America; 2 MitoCare Center for Mitochondrial Research, Department of Pathology, Anatomy and Cell Biology, Thomas Jefferson University, Philadelphia, Pennsylvania, United States of America; University of North Carolina School of Medicine, UNITED STATES

## Abstract

NF-κB is a major inflammatory response mediator in the liver, playing a key role in the pathogenesis of alcoholic liver injury. We investigated zonal as well as liver cell type-specific distribution of NF-κB activation across the liver acinus following adaptation to chronic ethanol intake and 70% partial hepatectomy (PHx). We employed immunofluorescence staining, digital image analysis and statistical distributional analysis to quantify subcellular localization of NF-κB in hepatocytes and hepatic stellate cells (HSCs). We detected significant spatial heterogeneity of NF-κB expression and cellular localization between cytoplasm and nucleus across liver tissue. Our main aims involved investigating the zonal bias in NF-κB localization and determining to what extent chronic ethanol intake affects this zonal bias with in hepatocytes at baseline and post-PHx. Hepatocytes in the periportal area showed higher NF-κB expression than in the pericentral region in the carbohydrate-fed controls, but not in the ethanol group. However, the distribution of NF-κB nuclear localization in hepatocytes was shifted towards higher levels in pericentral region than in periportal area, across all treatment conditions. Chronic ethanol intake shifted the NF-κB distribution towards higher nuclear fraction in hepatocytes as compared to the pair-fed control group. Ethanol also stimulated higher NF-κB expression in a subpopulation of HSCs. In the control group, PHx elicited a shift towards higher NF-κB nuclear fraction in hepatocytes. However, this distribution remained unchanged in the ethanol group post-PHx. HSCs showed a lower NF-κB expression following PHx in both ethanol and control groups. We conclude that adaptation to chronic ethanol intake attenuates the liver zonal variation in NF-κB expression and limits the PHx-induced NF-κB activation in hepatocytes, but does not alter the NF-κB expression changes in HSCs in response to PHx. Our findings provide new insights as to how ethanol treatment may affect cell-type specific processes regulated by NF-κB activation in liver cells.

## Introduction

The regenerative capacity of the liver has been widely studied in rodent models, particularly in the remnant liver after 70% partial hepatectomy (PHx) [1,2]. It is known that the response to an acute surgical challenge of PHx triggers a coordinated response of different cell types of the liver leading to the regulation of important liver functions [3,4]. Pro-inflammatory responses to PHx are associated with increased expression of numerous genes, activated by immediate early factors [5]. NF-κB is one such immediate early factor whose activity, induced by the pro-inflammatory cytokines, initiates a cascade of downstream regulatory processes [5,6]. It has been established that there is increased activation of NF-κB within the first 30 minutes following the surgery, which is maintained until approximately 4 hours [1,2,7,8]. Failure of NF-κB activation can result in reduced hepatocyte proliferation leading to impaired regeneration in the liver [9,10].

Chronic ethanol intake followed by PHx can cause dysregulation of the liver repair mechanisms potentially leading to aggravation of alcoholic liver disease [11,12]. Alcohol treatment increases apoptosis after PHx, and inhibits the proliferative activity of mature hepatocytes, causing a suppression of regeneration [13,14]. Chronic ethanol intake has been reported to induce a sustained increase in NF-κB activity in liver [12,15–17]. We tested whether such an increase was exhibited by hepatocytes in the chronic ethanol-adapted condition, and whether this sustained activity affected the liver response to PHx.

The liver shows zonally specific differences in mRNA and protein levels of various enzymes with preference towards either periportal or pericentral regions. This leads to zonal regulation of functions across the liver lobule, with the pericentral and periportal hepatocytes exhibiting complementary functions [18–20]. Such a spatial heterogeneity of gene regulation has an impact on the response to acute functional challenges, for example, in response to drug induced injury [21,22]. However, the spatial organization of the initial gene regulatory response to PHx is less clear. In addition, the potential zonal alterations in NF-κB activation due to ethanol adaptation have not been previously studied. Our study, for the first time, examined the zonal bias in NF-κB localization in liver with ethanol intake in hepatocytes at baseline and post-PHx states.

Recent single cell scale studies in a variety of tissues have uncovered the key functional role of cell-cell variations and the regulation of such heterogeneity in the tissue scale response [23–27]. Multiple studies show that liver regulatory programs are diverse within and across individual cells, even in the same cell types, in both rodents and humans [28]. Earlier studies showed induction of NF-κB activation in hepatocytes in response to regenerative stimuli [12,29,30]. Later studies reported Kupffer cells show the earliest and most marked NF-κB activation after liver injury [10,31]. Kupffer cell depleted liver tissue showed decreased NF-κB activation and delayed regeneration [32], suggesting that hepatocytes respond to a KC-derived stress signal with an activation of NF-κB. There is limited knowledge on the role of NF-κB in hepatic stellate cells (HSCs) in response to regeneration. Activation of HSCs is associated with nuclear translocation of NF-κB [33]. Activation of rat HSCs by cytokines TNF-α or IL1 triggers NF-κB activation and nuclear localization [34]. We focused our study on activation of NF-κB in hepatocytes and HSCs in response to ethanol adaptation following PHx. Our investigation was motivated in part by recent findings by Seki et al. that normal as well as Kupffer cell depleted mice showed nuclear translocation of NF-κB p65 in HSCs in response to acute LPS challenge [35]. This study showed HSC specific activation of NF-κB p65 as a result of LPS mediation. This has relevance in clinical studies since the NF-κB pathway is known to play a significant role in hepatic fibrogenesis [6]. Our studies led us to postulate that ethanol may enhance HSC activation in the chronic ethanol-adapted state as well as following PHx.

In this study, we asked whether the chronic ethanol-induced increase in NF-κB activation, as detected in tissue-scale extracts, is reflected uniformly across liver tissue. We were also interested in testing whether the increase in NF-κB activation at the ethanol-adapted baseline is associated with a robust NF-κB activation across the liver tissue in response to an acute challenge, such as PHx. We analyzed changes in nuclear localization of NF-κB p65 as a fraction of total cellular levels of this protein. We investigated the zonal bias in NF-κB localization with ethanol intake in hepatocytes at baseline and post-PHx states. We examined the activation profiles of NF-κB in single cells, particularly the cell type-specific NF-κB localization in hepatocytes and HSCs in the ethanol-adapted state, and assessed how acute perturbation due to PHx alters this distribution. We considered a time point at 3h post-PHx, the time scale around which NF-κB activity begins to decrease to assess the differences due to chronic adaptation to ethanol intake. It has been demonstrated that NF-κB activation post-PHx in chow-fed rats exhibits transient behavior between 1 and 6 hours, displaying an increase in the first two hours followed by a sharp decline within 4 hours, and once again increasing by 6 hours [7]. We focused on a post-surgical time point where the early activation response of NF-κB is close to its maximum in both dietary groups and the tissue is transitioning from the quiescent to the primed state. In each case, we investigated the potential spatial heterogeneity and zonal alterations in NF-κB localization.

## Materials and Methods

### Ethics Statement

All animal protocols were carried out in strict adherence to the guidelines from the Institutional Animal Care and Use Committees (IACUC) of Thomas Jefferson University and conform to the principles of USA regulations. The protocol was approved by the Committee on the Ethics of Animal Experiments of Thomas Jefferson University.

### Animals

In this study, we used four adult male Sprague-Dawley rats (Rattus norvegicus). Rats were held in single occupancy cages in a climate controlled, 12-hr day/night cycle in accordance with acceptable animal handling practices at TJU.

### Diets

Rats were fed using the Lieber-DeCarli feeding model to induce chronic ethanol exposure [36]. The animals were fed a liquid diet with 36% of total calories provided by ethanol (6.2% v/v); littermate control rats were pair-fed with liquid diet in which ethanol was replaced isocalorically by carbohydrate (maltose-dextran) for 6–8 weeks (ethanol-fed, N = 2; carbohydrate, N = 2).

### Partial Hepatectomy

In accordance with ethical practices, the animals were anesthetized using isoflurane at 5% concentration in a dedicated anesthesia chamber prior to and during the surgery. The 70% partial hepatectomy (PHx) model of liver regeneration was used in which the left lateral and medial lobes (LLM) were removed, rapidly frozen and stored as a baseline sample from each rat. The surgery was performed following the standard protocol with modifications to comply with current IACUC guidelines [37]. Following surgery, but while under anesthesia, animals received 1 ml intraperitoneal (IP) injections of lactated ringers solution (LRS) pre-warmed to 37°C to ensure adequate hydration following surgery. The anesthesia was removed at the end of the surgical procedure and the animal returned to its cage. After the animal recovered and started moving, it was monitored closely for signs of distress for the first hour (and for 10 minutes hourly thereafter) while it was kept in its cage with access to regular diet (*ad libitum*) until the time of sacrifice. Cages were kept warm with heating lamps for the first three hours following surgery. Three hours after surgery, the animal was again anesthetized, the original incision was reopened and the remnant liver was harvested. The animal was euthanized by cervical dislocation and opening of the chest cavity. Each collected liver sample was embedded in OCT and flash frozen in liquid nitrogen and stored at temperature of -80°C. The frozen tissue samples were then sectioned and collected in this fashion for both control and ethanol-fed animals. All surgeries were performed between 8AM - 11AM in order to minimize circadian rhythm effects.

### Multiplex Immunofluorescence Staining

**Fig 1:**
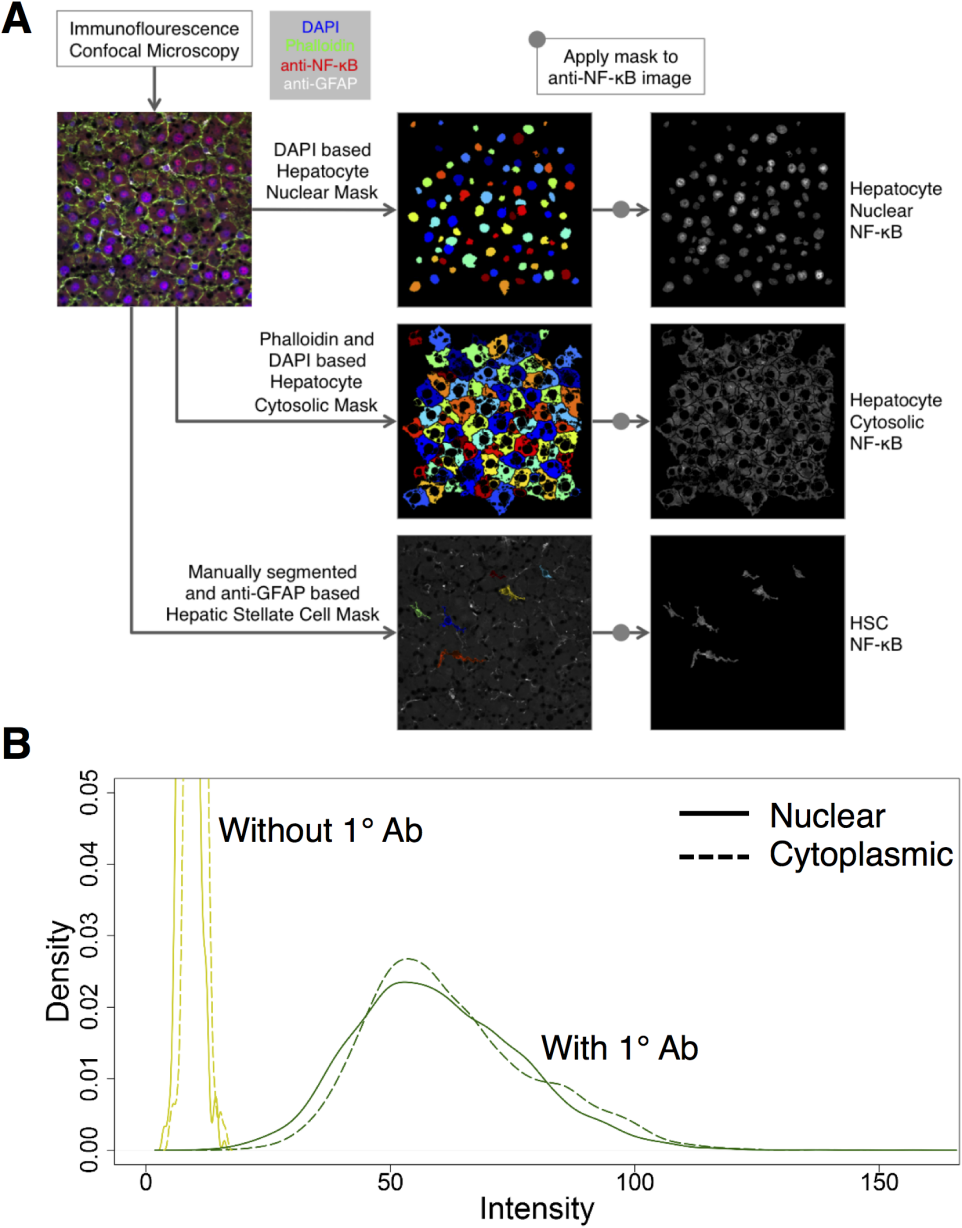
(A) Schematic of the immunohistochemistry and image analysis workflow. Frozen rat liver tissue was stained for DAPI, Phalloidin, GFAP and NF-κB (refer to Materials and Methods: Multiplex Immunofluorescence Staining). Images were acquired using confocal microscopy. These images were analyzed by automated image segmentation techniques involving a combination of thresholding, masking and intensity measurement modules in the CellProfiler software. The intensity data obtained from CellProfiler was analyzed through R Programming. **(B)** Negative control analysis. The curves in yellow mark the nuclear (solid) and cytoplasmic (dotted) intensity distributions from slides stained without including primary antibodies. The curves in green show the nuclear (solid) and cytoplasmic (dotted) intensity distributions of the baseline carbohydrate tissue. The negative control group showed negligible intensity signal.

Our immunofluorescence protocols were derived from established frozen tissue staining protocols that have been established to work with sections of 10μm thickness, usable for confocal microscopy [38]. Frozen tissue blocks were sectioned at 10μm and stored at -80°C prior to use. Sections were fixed in 4% paraformaldehyde (Electron Microscopy Sciences, Hatfield, PA) and permeabilized using Triton X-100 (LabChem, Zelienople, PA) at a concentration of 0.01% for 15 minutes. Permeabilization and subsequent steps were carried out at room temperature and typically separated by 3 washes of 5 minutes each in PBS 1X (Fisher Bioreagents), unless specified otherwise. Sections were then protein blocked using 3% Normal Goat Serum (Vector Laboratories, Burlingame, CA) for 35 minutes. The multiplex mixture of primary antibodies contained anti-NF-κB (Ab7970-1, AbCam, Cambridge, MA; 1:100) and anti-GFAP (Ab4674, AbCam; 1:1000), while the multiplex mixture of secondary antibodies was prepared at dilutions of 1:400 for AlexaFluor 647 (A21244, Life Tech, Carlsbad, CA, targeting anti-NF-κB) and 1:1500 for Cy3 (103-165-155, Jackson Immunoresearch, West Grove, PA, targeting anti-GFAP). These antibodies have been employed extensively in previous studies to specifically detect the respective proteins in western blots and immunohistochemistry assays, including using a p65 knock out model (e.g., anti-NF-κB–[39–41]; anti-GFAP–[42]). Negative controls in the absence of the primary antibody showed negligible signal intensity as evidenced by the intensity distribution shown in Fig 1B. Slides were incubated overnight at room temperature with the primary multiplex mixture followed by 3 washes of 10 minutes each. This was followed by incubation with the secondary antibody mixture for 1 hour and 45 minutes followed by another set of 3 washes of 10 minutes each. After staining for antibodies, DAPI (D9542, Sigma) and AlexaFluor-488-conjugated Phalloidin (A12379, Life Tech) were applied together and allowed to incubate for 30 minutes after which ProLong® Gold Antifade (Life Tech) solution was applied. The slides were mounted with coverslips and allowed to cure overnight at room temperature, before microscopic examination.

### Confocal Microscopy

Images were acquired using a Zeiss LSM 780 mounted on a Zeiss axio observer inverted microscope. The 405 nm, 488 nm, 561 nm and 633 nm lasers were used, having been set with a unique and high-contrast color for each channel. The images were captured using the Zeiss ZEN 2011 software package associated with the LSM 780. Within ZEN 2011, the detectors were set at standard available ranges for specified dyes with little modification; pixel resolution was set at 1024x1024, at 8-bit color depth with a line scan and an averaging setting of 4. Prior to acquisitions, saturations and zeros were set using the range indicator.

For imaging, two tracks were created with two lasers each, in such a way that there would be no interference between laser channels. The 405 nm and 561 nm lasers were added into track 1 and the 488 nm and 633 nm lasers were added into track 2. The 405 nm laser was used to capture DAPI, the 488 nm was used to illuminate the AlexaFluor 488-Phalloidin, the 561 nm was aimed at exciting the Cy3-conjugated secondary and the 633 nm was used to target the AlexaFluor 647-conjugated secondary. We examined the total amount of NF-κB in the nucleus–as measured by the nuclear co-localization of NF-κB and DAPI. This was calculated as the nuclear fraction, i.e., the fraction of total cell fluorescence intensity coming from the nucleus.

### Digital Image Analysis

The digital image analysis tool CellProfiler was used to develop a novel automated image analysis pipeline [43]. For each multiplex immunostained image set, DAPI gray scale image was used to develop a mask corresponding to the hepatocyte nuclei. This mask was applied to the NF-κB image to calculate hepatocyte nuclear NF-κB intensity values. Similarly, a combination of Phalloidin and DAPI images were used to develop a hepatocyte cytosolic mask, which was then applied to the NF-κB image to calculate hepatocyte cytosolic NF-κB intensity values. The HSCs were manually segmented based on anti-GFAP image and this mask was applied to the NF-κB image to calculate whole HSC NF-κB intensity values. The acquired images were first converted into a JPEG format in greyscale for each channel. These images were analyzed by automated image segmentation techniques involving a combination of thresholding, masking and intensity measurement modules in the CellProfiler software. The novel CellProfiler pipeline was developed specifically for our image analysis. The pipeline consisted of several modules serving to perform one or more image segmentation-related functions such as thresholding, object identification, masking, intensity measurement and exporting into spreadsheets.

### Image Data Analysis

The intensity data obtained from the automated image analysis were analyzed using the R programming language [44]. The scripts written in R (available upon request) were designed to normalize the data, calculate distributions, and perform permutation analysis and multivariate analyses.

### Normalization

The object-based mean intensity measures were used for our analyses. For each image, a mean intensity was calculated using the total number of observations (cellular objects) per image. A weighted global average was taken as the sum of all image-specific mean intensities, divided by the number of images and weighted according to the number of observations per image. This global mean intensity value was then divided by image-specific mean intensity to calculate a normalization factor for each image, which was then applied to all observations of the corresponding image.

### Distributions

The normalized nuclear and cytoplasmic data were used to calculate a nuclear fraction per cellular object. The cumulative distributions were then calculated for the entire data set.

Based on the fact that a hepatocyte nucleus is approximately 6% of the whole cell volume [45], we calculated that the area occupied by the nucleus is approximately 15% of that of the whole cell in a two dimensional histopathological and optical section that is at the equatorial level of the nucleus considered as a sphere. Accordingly, we scaled the nuclear and cytoplasmic intensities, respectively, to calculate the nuclear fraction as:

$$\text{Nuclear Fraction}\;=\frac{\left(0.15\times I_{N}\right)}{\left(0.15\times I_{N}\right)+\left(0.85\times I_{C}\right)}$$

Where *I*_*N*_ is nuclear intensity and *I*_*C*_ is cytoplasmic intensity.

### Permutation Testing and Statistics

Data were randomized and distributions were calculated, in order to evaluate if the levels of difference seen between randomized data groups were similar to that of the original arrangement of our data. This method of testing aided in confirming that any difference observed in the nuclear fractions between groups was not due to random chance. We performed 10,000 iterations of randomized permutations.

### Real-Time PCR

Whole-liver tissue samples were homogenized and RNA extracted using an animal tissue RNA purification kit (Norgen, ON, Canda). RNA extracts were tested for overall RNA quantity using a NanoDrop spectrophotometer (NanoDrop, Wilmington, DE). Total RNA was converted to cDNA and pre-amplified prior to real-time qPCR analysis using the Biomark platform (Fluidigm, San Francisco, CA). The data were normalized by median-centering within each sample, followed by median-centering within genes. Primers used for PCR analysis are available in S1 Table.

### Statistical Analysis

The statistical significance of the differences in the average nuclear fraction values were evaluated using a Wilcoxon Signed-Rank Test, using the function *wilcox*.*test* in the R statistical analysis platform [44]. The gene expression data were analyzed using a one-way ANOVA model considering one factor on four levels (Carbohydrate baseline, Carbohydrate PHx, Ethanol baseline, Ethanol PHx). Comparisons between each group were performed using *post hoc* Tukey Honest Significant Difference contrast testing. A p-value threshold of 0.05 was utilized for statistical significance. We employed the *aov* and *TukeyHSD* functions in the R statistical analysis platform [44].

## Results

We developed an integrative workflow consisting of multiplex immunofluorescence staining, digital image analysis and statistical distributional analysis of immunofluorescence data. A schematic of the integrated analysis pipeline is shown in Fig 1A. Our approach was motivated by previous studies that utilized a combination of staining techniques and image analysis to investigate hepatic pathophysiology [46,47]. We evaluated NF-κB activation as reflected by the nuclear localization levels computed as the fraction of total cellular intensity localized to the nucleus. We utilized this metric for comparison (1) across cells to assess lobular as well as liver cell-type specific distributions, and (2) across conditions to evaluate the effects of chronic ethanol intake and PHx. Our unique image analysis approach enabled us to automatically identify cell boundaries and analyze distribution of NF-κB localization segregated between nucleus and cytoplasmic compartments of hepatocytes and HSCs. We analyzed the intensities from each object and evaluated the differences in NF-κB localization and their zonal preference between various treatment groups.

### Adaptation to chronic ethanol consumption shifts the distribution of NF-κB localization

#### Variation in hepatocytes

**Fig 2:**
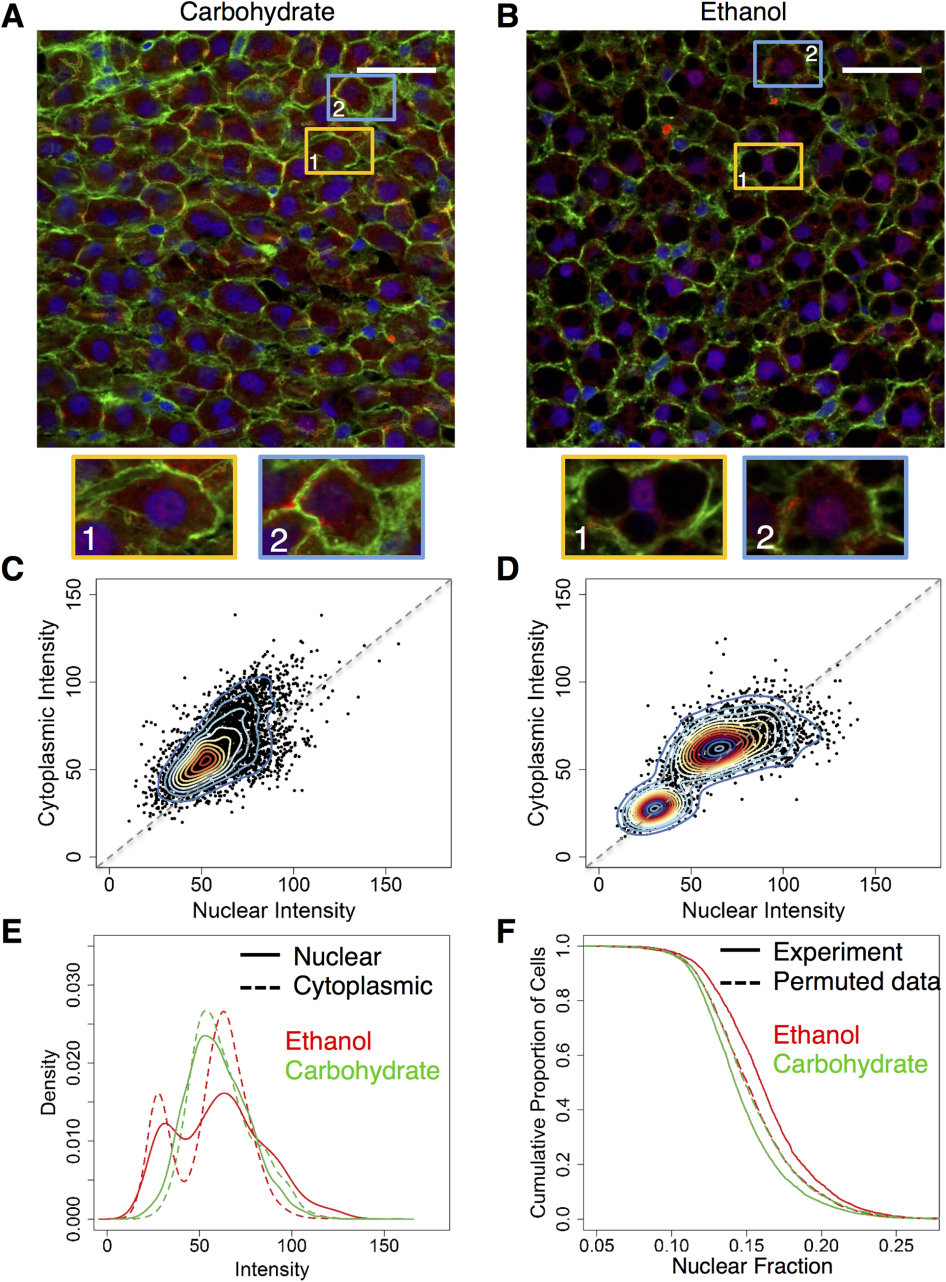
NF-κB localization in the baseline ethanol-adapted condition (N = 2 rats/condition, N = 6871 cells total) **(A and B)** Merged images of tissue stained with Alexa Fluor-488-labeled phalloidin (green) and Alexa Fluor-647-labeled antibody for NF-κB (red) and nuclei that were stained with DAPI (blue). The regions highlighted by the orange rectangles, indicated by the numbering-1, shows a representative cell from each image with a visually high level of nuclear co-localization and the regions in the blue rectangles, indicated by the numbering-2, shows a representative cell from each image having a visually low nuclear co-localization. Scale bar: 40 μm. **(C and D)** Two-dimensional kernel density estimations of baseline carbohydrate (C) and ethanol (D) groups. The ethanol group shows two distinct populations of cells, one in a higher range of intensities (numbered in red-2) than the other. The carbohydrate group shows one population of cells. **(E)** Distributions of nuclear (solid) and cytoplasmic (dotted) intensities of both baseline groups. Ethanol (red) shows a bimodal distribution, with second subpopulation showing a shift towards the higher extreme when compared to the carbohydrate (green) group. **(F)** Nuclear fractions of intensity of both baseline groups as well as representative permuted data groups. The ethanol group (red) of cells showed a significantly higher nuclear fraction of NF-κB than the carbohydrate group (green) of cells. The permuted data (dotted lines) iterated over 10000 times did not report as large a difference between the two groups, as seen in the original data. (Total no of cells: Baseline Ethanol: 2938, Baseline Carbohydrate: 3941).

Our results showed that NF-κB localization in the cells spans a wide range of intensities across all treatment conditions, including in response to chronic ethanol consumption. In order to understand the full extent of the diversity of NF-κB localization in the hepatocytes, we first carried out a visual examination to find explicit differences between the ethanol-adapted and the pair-fed control (carbohydrate) diet groups. Ethanol-adapted rats showed lipid accumulation in the tissue, which was absent in livers from pair-fed controls (Fig 2A and 2B). However, both diet groups showed a mixed population of cells, exhibiting variability in nuclear co-localization, i.e., both groups consisted of cells with high and low nuclear localization of NF-κB (Fig 2A and 2B).

We observed variability in NF-κB localization profiles, even within the same tissue and field of view. The kernel density of the baseline carbohydrate group, though widespread, showed only a single population with nuclear and cytoplasmic intensities varying almost linearly (Fig 2C). However, the bivariate kernel density diagram for the chronic ethanol group revealed two distinct cell populations, with one of the population showing relatively higher levels of both nuclear and cytoplasmic intensities (Fig 2D). The behavior of both diet groups indicated that the liver cells exhibit varying levels of localization and that some cells may not contain detectable levels of nuclear NF-κB. There was no clear spatial clustering of the two subpopulations across the liver tissue, indicating that the bimodal NF-κB response to ethanol may not be due to differences in local microenvironment. However, a shift from a higher cytoplasmic intensity to slightly higher nuclear intensity was observed in the ethanol group, particularly for the second sub population with higher intensity (Fig 2C and 2D). This was evident in the shift in distribution of the two cell populations from the ethanol group compared to that of the carbohydrate group, with one subpopulation showing lower intensity distribution and another showing a shift towards higher intensity levels (Fig 2E). We utilized the nuclear and cytoplasmic intensities to compute the fraction of the whole-cell intensity that is localized to the nucleus in each cell, i.e., the nuclear fraction. The ethanol group showed a statistically significant increase in the average nuclear fraction compared to the carbohydrate group (S1 Fig). Analysis of the nuclear fraction distribution revealed a shift in NF-κB localization distribution, suggesting an increase in NF-κB activity in many cells after adaptation to chronic ethanol intake (Fig 2F). This shift towards a higher nuclear fraction largely occurred in the low and middle range of the distribution and was less pronounced towards higher values. We interpret these results as suggesting that the differences between the diet groups is not due to a cell subpopulation that shows very high NF-κB signaling at the baseline in the ethanol group. We performed permutation testing on our intensity values over 10,000 iterations to evaluate whether the differences seen in our data could have occurred by random chance (p < 0.01). Our experimentally observed difference was a significant outlier relative to the random chance distribution (Fig 2F).

#### Zonal differences between NF-κB localization and distribution

**Fig 3:**
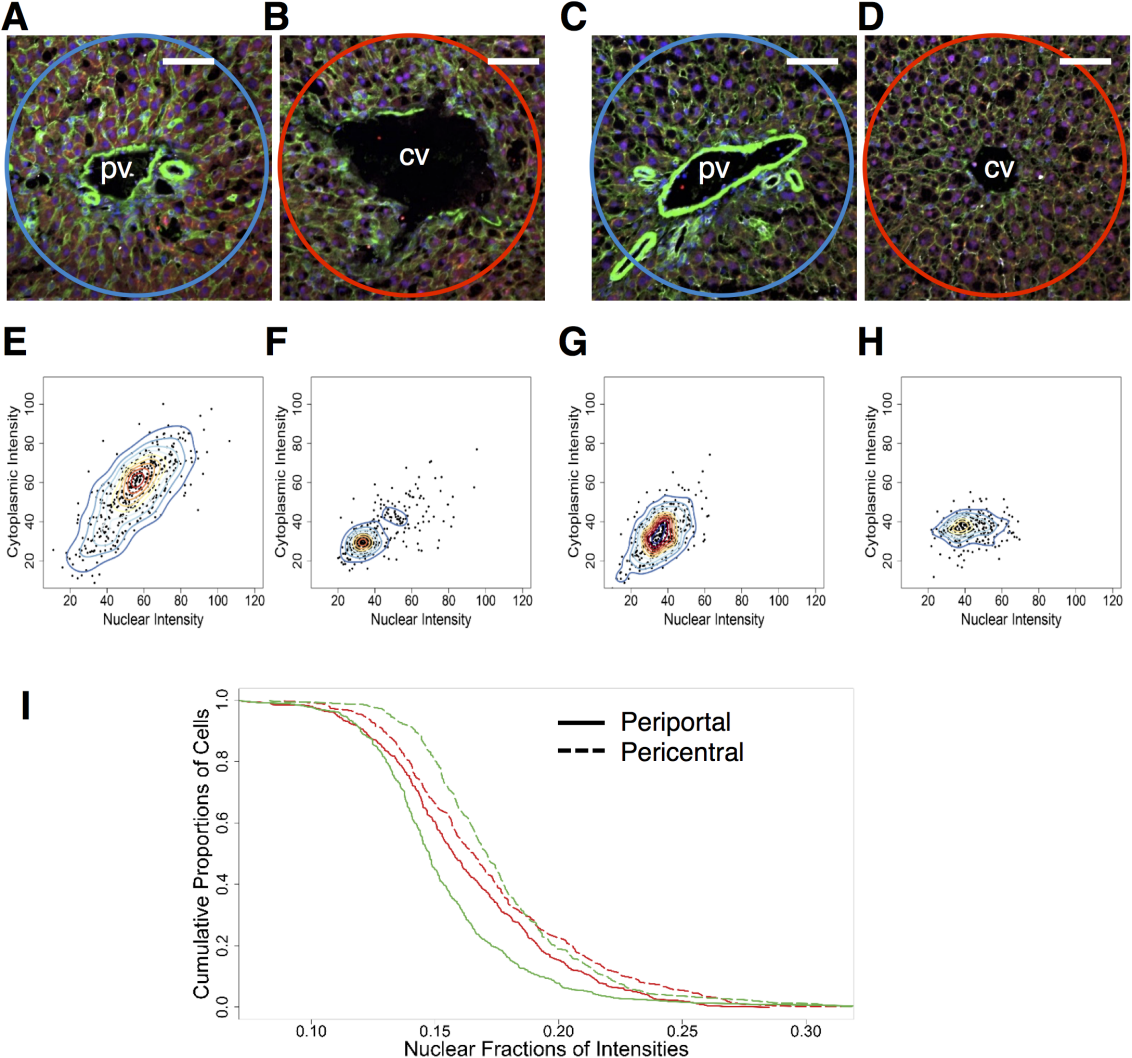
Zonal alteration of NF-κB localization in the ethanol-adapted condition (N = 2 rats/condition, N = 1490 cells total). **(A** and **B)** Representative images of portal triads of baseline carbohydrate (A) and ethanol (B) groups. The periportal region is highlighted by the blue circle. Scale bar: 40 μm. **(C** and **D)** Representative images of central veins of baseline carbohydrate (C) and ethanol (D) groups respectively. The pericentral region is highlighted by the red circle. **(E** and **F)** Two-dimensional Kernel Density distribution of periportal (E) pericentral (F) cells from carbohydrate-fed baseline animal groups. Compared to their periportal counterparts, the cells in periportal group are much more widespread spanning a larger range of intensities than their pericentral counterparts. **(G** and **H)** Two-dimensional kernel density distribution of periportal (E) pericentral (F) cells from ethanol adapted animal groups. **(I)** Nuclear fraction of intensities of periportal and pericentral cells of carbohydrate (green) and ethanol (red) baseline groups. Solid lines represent periportal nuclear fractions, dashed lines represent pericentral nuclear fractions. (Total no of cells—Baseline: Ethanol periportal: 409, Ethanol pericentral: 293, Carbohydrate periportal: 440, Carbohydrate pericentral: 348).

The zonal differences across the liver acinus, although widely studied in the context of genes and their expression, have not been fully investigated in the context of transcription factors and their activation. The above experiments involved hepatocytes taken specifically from the midzonal region, which is well outside the perimeter of what we considered in the periportal and pericentral groups. We therefore investigated the zonal differences in NF-κB localization due to adaptation to ethanol. We analyzed multiplex immunofluorescence images tiled around and including the portal and central veins in order to explore the possibility of zonal alterations in NF-κB localization. All of the cells within the periportal and pericentral regions were selected based on their coordinates and were analyzed further to detect putative zonal alterations in NF-κB localization (Carbohydrate–Fig 3A and 3B; Ethanol–Fig 3C and 3D). The kernel density plots comparing periportal and pericentral zones showed only one cell population in each case (Carbohydrate–Fig 3E and 3F; Ethanol–Fig 3G and 3H). Periportal cells from both diet groups showed a wider range of intensities than the pericentral cells (Fig 3E and 3G). Within the carbohydrate group, the observed NF-κB levels spanned a wider range of nuclear and cytoplasmic intensity in the periportal region than in the pericentral region (Fig 3E and 3F). In the ethanol group, however, the cytoplasmic intensity of the pericentral cells spanned over a smaller range of intensities compared to the periportal cells (Fig 3G and 3H).

Analysis of the nuclear fraction distribution also revealed that the pericentral cells of both diet groups showed a shift towards higher nuclear localization than the periportal cells (Fig 3I). However nuclear fraction calculation revealed that carbohydrate group showed the highest levels of NF-κB localization in the pericentral region, compared to the periportal region as well as in comparison to both regions in the ethanol group (Fig 3I). This was consistent in the nuclear fraction estimates in ethanol diet groups as a shift in distribution towards higher NF-κB localization in the pericentral cells compared to periportal cells (Fig 3I). However, within this group, periportal and pericentral zones did not show a significant difference in the whole cell or nuclear NF-κB intensity.

In summary, for both diet groups the pericentral cells displayed higher NF-κB nuclear fraction profiles than their corresponding periportal cells that were distributed over a wider range of NF-κB localization levels (Fig 3I). Analysis of the nuclear fraction revealed that the difference in the NF-κB localization between the pericentral and periportal cells appeared to be smaller after adaptation to chronic ethanol consumption in comparison to the carbohydrate control group.

#### Variation in hepatic stellate cells

**Fig 4:**
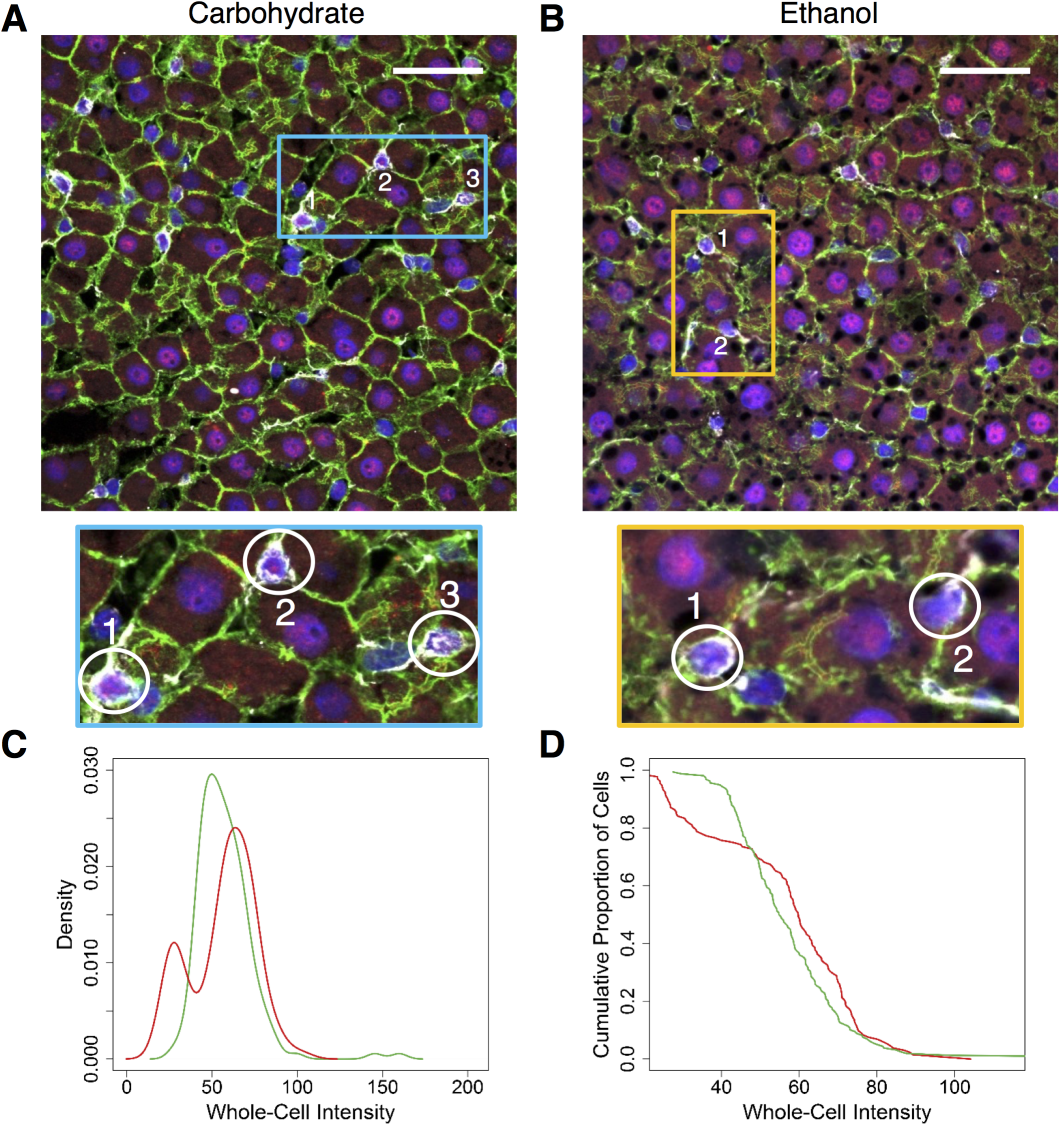
Whole-cell levels of NF-κB in the HSCs in the ethanol-adapted condition (N = 2 rats/condition, N = 284 cells total). **(A and B)** Merged images of tissue stained with Alexa Fluor-488-labeled Phalloidin (green) and Alexa Fluor-647-labeled antibody for NF-κB (red), nuclei which were stained with DAPI (blue) and the HSCs, which were stained with Cy3 labeled-antibody for GFAP (HSC marker). The highlighted regions in the blue and orange rectangles show typical HSCs, magnified further. Scale bar: 40 μm. **(C)** Distributions of whole-cell intensities measured from the HSCs of both baseline groups. The ethanol group shows two populations, with the second one showing a higher level of whole-cell NF-κB than the carbohydrate group. **(D)** Cumulative distributions of the whole-cell intensities of the HSCs from both groups. Close to 80% of the HSCs from the ethanol group show higher whole-cell levels of NF-κB than carbohydrate group. (Total no of cells: Baseline Ethanol: 132, Baseline Carbohydrate: 152).

The diversity exhibited by the hepatocytes at the baseline was also observed in hepatic stellate cells in response to chronic ethanol consumption. We manually identified and segmented HSCs as described in the Methods based on GFAP immunofluorescence (Fig 4A and 4B). However, in the case of stellate cells, the nuclear fraction calculation was not sufficiently reliable as most of the visual field of each HSC was comprised of the nucleus (Fig 4A and 4B). To overcome this issue, we quantified the whole-cell intensity to analyze NF-κB levels in HSCs. While it was difficult to reliably segregate cytosolic and nuclear fractions in HSCs, we note that the data largely corresponds to the nuclear NF-κB levels and have interpreted the results as such. The HSCs of the ethanol group showed two distinct populations of whole-cell NF-κB intensity distributions, with the second population showing higher whole-cell levels of NF-κB than the carbohydrate group (Fig 4C). We found that that almost 80% of the HSCs from the ethanol group showed higher whole-cell levels of NF-κB than the carbohydrate group (Fig 4D). Previous immunofluorescence studies revealed the presence of NF-κB p65 in the nuclei of activated HSCs [34]. Consistent with these findings, our results showed an activation of NF-κB in HSCs due to ethanol intake.

### Deficient NF-κB response in the ethanol-adapted group at 3h post-PHx

#### Variability in response to PHx in hepatocytes

**Fig 5:**
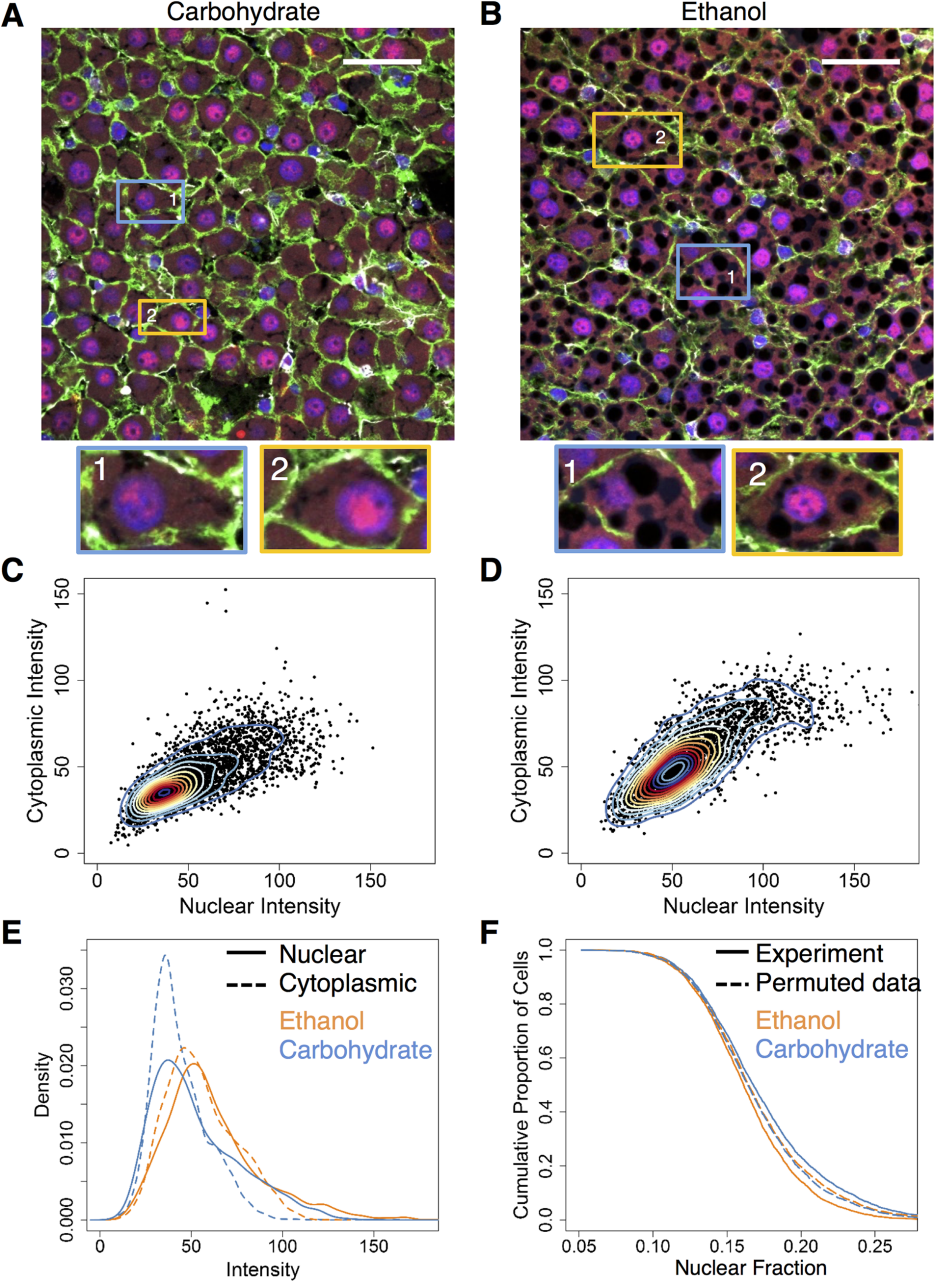
NF-κB localization post partial hepatectomy (N = 2 rats/condition, N = 6860 cells total). **(A and B)** Merged images of tissue stained with Alexa Fluor-488-labeled Phalloidin (green) and Alexa Fluor-647-labeled antibody for NF-κB (red) and nuclei, which were stained with DAPI (blue). The regions highlighted by the blue rectangles, indicated by the numbering-1, shows a representative cell from each image with a visually low level of nuclear co-localization and the regions in the orange rectangles, indicated by the numbering-2, shows a representative cell from each image having a visually high nuclear co-localization. Scale bar: 40 μm. **(C and D)** Two-dimensional kernel density estimations of post-PHx carbohydrate (C) and ethanol (D) groups. **(E)** Distributions of nuclear (solid) and cytoplasmic (dashed) intensities of carbohydrate (dark blue) and ethanol (orange) groups at 3h post-PHx. Ethanol group shows a shift towards the higher extreme when compared to the carbohydrate group. **(F)** Nuclear fractions of intensity of both baseline groups as well as representative permuted data groups. The cells of carbohydrate group show a significantly higher nuclear fraction of NF-κB than the cells of ethanol group. The permuted data iterated over 10000 times did not report as large a difference between the two groups, as seen in our original data. (Total no of cells: post-PHx Ethanol: 3221, post-PHx Carbohydrate: 3639).

NF-κB activation shows a rapid transient increase within the first two hours after PHx but declines by 4h post-PHx [7]. We chose to characterize sub-cellular NF-κB localization at the 3-hour time point, before its expression is down regulated. Our interest was to determine the effect of PHx on NF-κB intensity distribution, as well as to analyze how adaptation to ethanol intake affects the response of NF-κB nuclear localization at 3h post-PHx. We found NF-κB to be actively relocated to the nucleus in the carbohydrate diet group as a unimodal cell population revealed by the kernel density estimate (Fig 5C). The carbohydrate group showed higher nuclear intensity of NF-κB following PHx, consistent with expected activation of NF-κB during this time period. However, in the case of the ethanol group, the bimodal distribution observed at the baseline ethanol-adapted condition was not clearly observed post-PHx. Instead, our analysis identified a single cell population distributed over a higher range of intensities in the ethanol-adapted hepatocytes. Comparison within the ethanol diet group before (Fig 2D) and after PHx (Fig 5D) revealed an increase in NF-κB localization post-PHx. However, there is a possibility of a smaller scattered subpopulation of cells with higher NF-κB intensity, which is not clearly recognized as an independent population in the kernel density analysis. Another possibility for the unimodal distribution is the activation of NF-κB expression in the previously observed cells of the smaller subpopulation (Fig 2D) with no NF-κB localization, to a single merged population of higher intensity (Fig 5D). We speculate that in the ethanol-adapted state, only a fraction of cells may remain responsive to ethanol and show higher level of NF-κB activation. However, when additionally subjected to the acute challenge of partial hepatectomy, the effects are broad-based with a larger fraction of cells being responsive to the signals elicited by partial hepatectomy and hence the subpopulation with lower NF-κB levels disappears. The nuclear and cytoplasmic intensity distributions of both groups did not show significant differences, but both cytoplasmic and nuclear NF-κB intensities were higher after chronic adaptation to ethanol intake (Fig 5E).

In contrast to the intensity distributions, the nuclear fractions displayed an opposite trend. The ethanol group showed a statistically significant decrease in the average nuclear fraction compared to the carbohydrate group, at 3h post-PHx (S1 Fig). Analysis of the nuclear fraction distribution showed a shift towards higher nuclear fraction in the carbohydrate group as compared to the ethanol group (Fig 5F). This shift was more pronounced towards the higher nuclear fraction levels, suggesting that the cell subpopulation with robust NF-κB signaling in the carbohydrate group is attenuated in the ethanol group. We performed a permutation analysis to test whether the observed difference in nuclear fraction distributions was due to a random chance. The cytoplasmic and nuclear data were permuted over 10000 iterations and the resulting nuclear fraction distributions were re-calculated. In contrast to our experimental findings, the permuted data failed to show significant differences between the diet groups (Fig 5F) (p < 0.01), indicating that the differences in NF-κB localization observed experimentally are not likely due to statistical random chance. We compared the nuclear fractions of both the ethanol and carbohydrate groups for changes between baseline and post-PHx.

#### Zonal differences between NF-κB localization and distribution post-PHx

**Fig 6:**
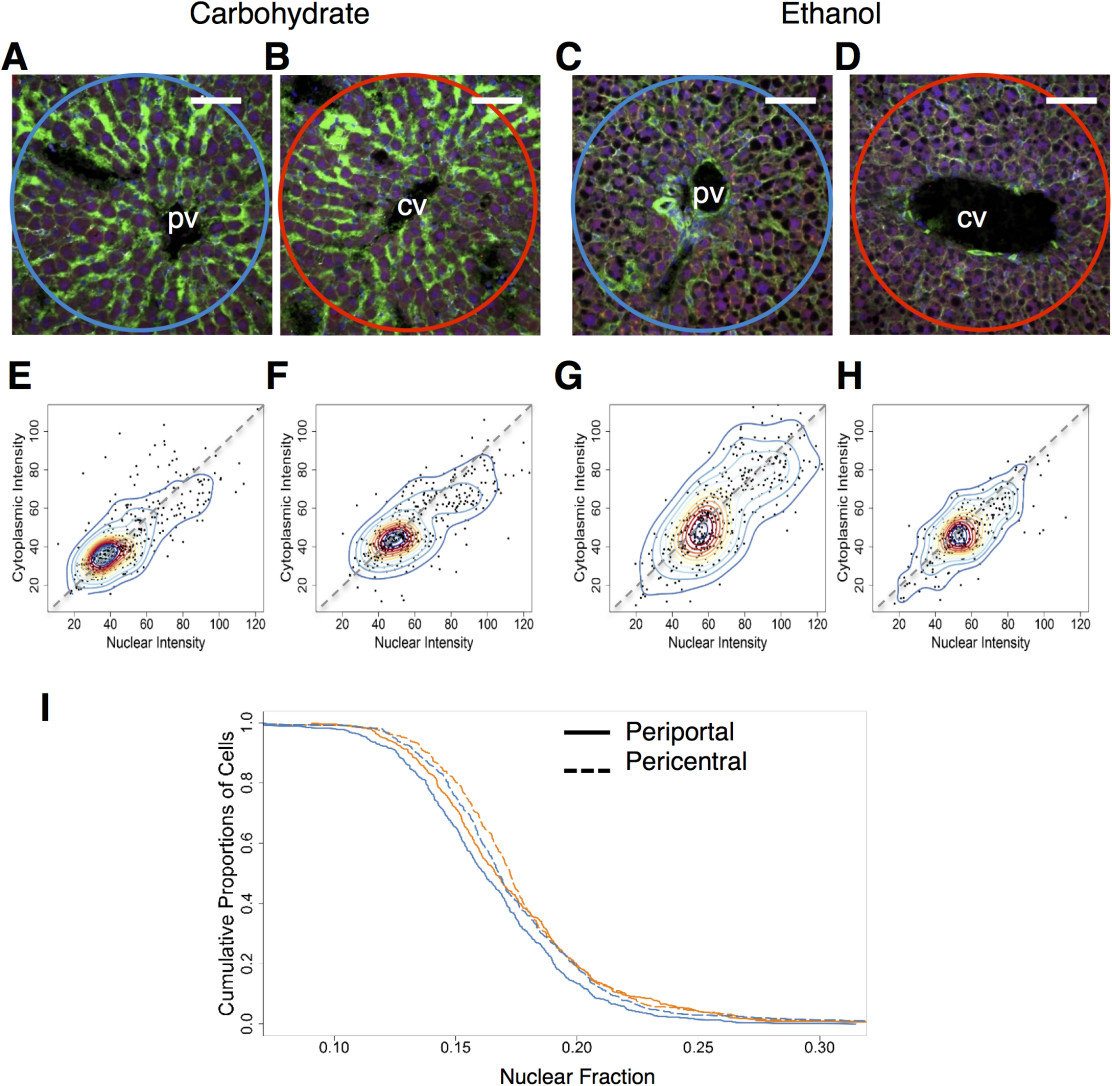
Zonal alteration of NF-κB localization post partial hepatectomy. (N = 2 rats/condition, N = 1535 cells total). **(A and B)** Representative images portal triads of post-PHx Carbohydrate (A) and Ethanol-adapted (B) groups, respectively. The region highlighted by the blue circles is the periportal region. Scale bar: 40 μm. **(C and D)** Representative images of central veins of post-PHx carbohydrate (C) and adapted (D) groups respectively. The region highlighted by the red circle is the pericentral region. **(E and F)** Two-dimensional kernel density distribution of periportal (E) pericentral (F) cells from carbohydrate-fed post-PHx animal groups. **(G and H**) Two-dimensional kernel density distribution of periportal (E) pericentral (F) cells from ethanol-fed post-PHx animal groups. **(I)** Nuclear fraction of intensities of periportal (solid) and pericentral (dotted) cells of carbohydrate (dark blue) and ethanol (orange) post-PHx groups. (Total no of cells—post-PHx: Ethanol periportal: 418, Ethanol pericentral: 295, Carbohydrate periportal: 416, Carbohydrate pericentral: 406).

We sought to examine whether the zonal patterns of NF-κB localization that were seen in the baseline groups varied in response to partial hepatectomy. In order to investigate the potential zonal alterations in NF-κB localization, we analyzed multiplex immunofluorescence images tiled around and including the portal and central regions (Carbohydrate–Fig 6A and 6B; Ethanol–Fig 6C and 6D). The periportal and pericentral cells of post-PHx carbohydrate groups showed similar range of intensity distribution. They were spread across a higher range of intensities than their baseline counterparts (Fig 6E and 6F). We observed a similar distribution in the ethanol group. In ethanol fed animals, the periporal cells spanned a wider range of NF-κB intensity compared to the pericentral cells (Fig 6G and 6H). However, this distinction was not evident in the nuclear fraction distributions. The NF-κB nuclear fraction distributions revealed no clear distinction between any of the post-PHx groups (Fig 6I).

#### Variation in HSCs post-PHx

**Fig 7:**
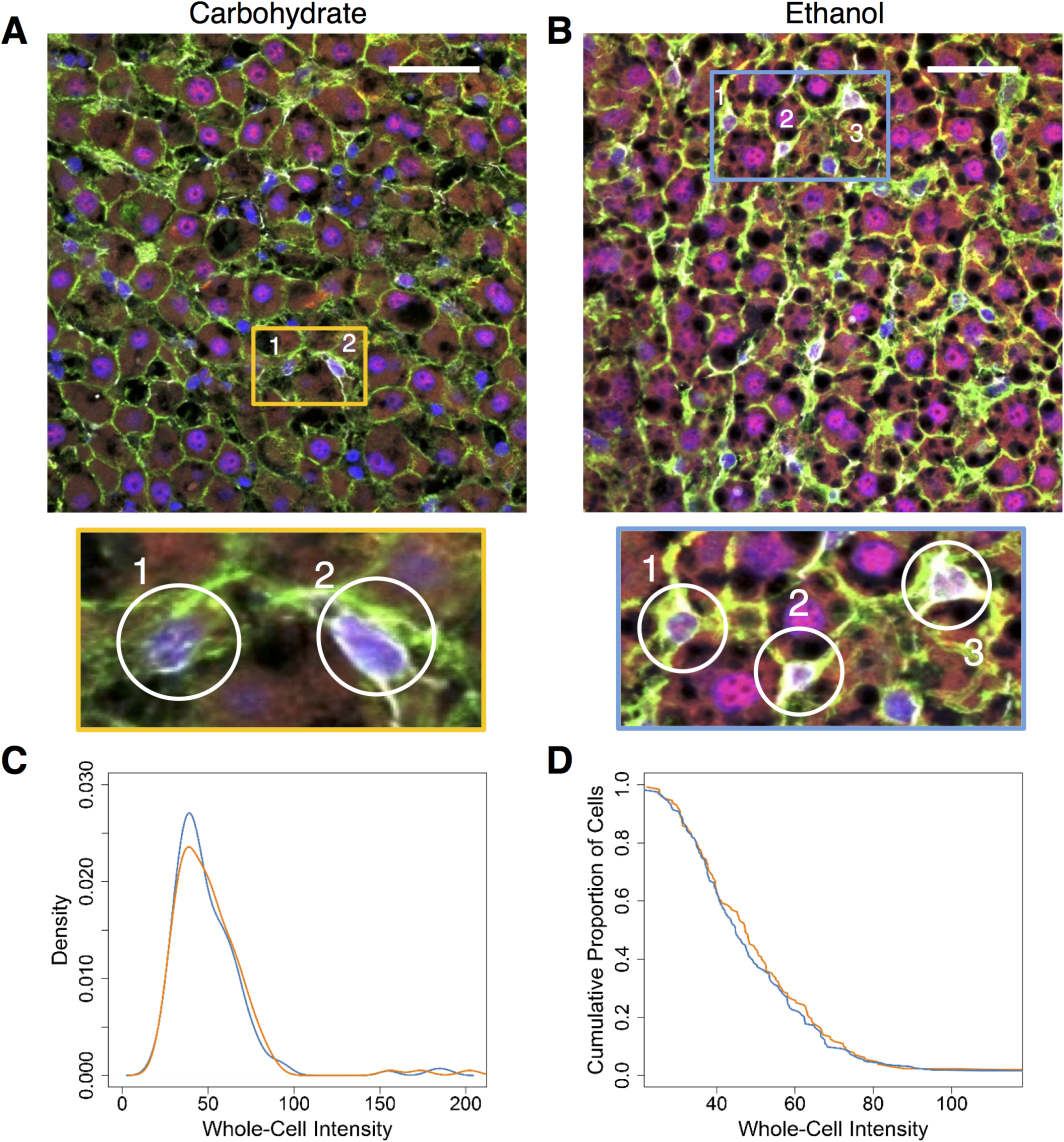
Whole-cell levels of NF-κB in the HSCs post partial hepatectomy (N = 2 rats/condition, N = 291 cells total). **(A and B)** Merged images of tissue stained with Alexa Fluor-488-labeled phalloidin (green) and Alexa Fluor-647-labeled antibody for NF-κB (red), nuclei which were stained with DAPI (blue) and the HSCs, which were stained with Cy3 labeled-antibody for GFAP (HSC marker). The highlighted regions in the blue and orange rectangles show typical HSCs, magnified further. Scale bar: 40 μm. **(C)** Distributions of whole-cell intensities measured from the HSCs of both post-PHx groups. There does not seem to be a significant distinction in the NF-κB localization between the carbohydrate group (dark blue) and the adapted group (orange). **(D)** Cumulative distributions of the whole-cell intensities of the HSCs from both groups. (Total no of cells: post-PHx Ethanol: 128, post-PHx Carbohydrate: 163).

We analyzed the distribution of whole-cell NF-κB intensities in HSCs of the post-PHx groups (Fig 7A and 7B). The distribution spanned a wide range of intensities in HSCs and was found to be overlapping in both ethanol and carbohydrate diet groups, without clear distinction in the levels (Fig 7C and 7D).

#### Overall comparison between ethanol-adapted and post-PHx conditions

**Fig 8:**
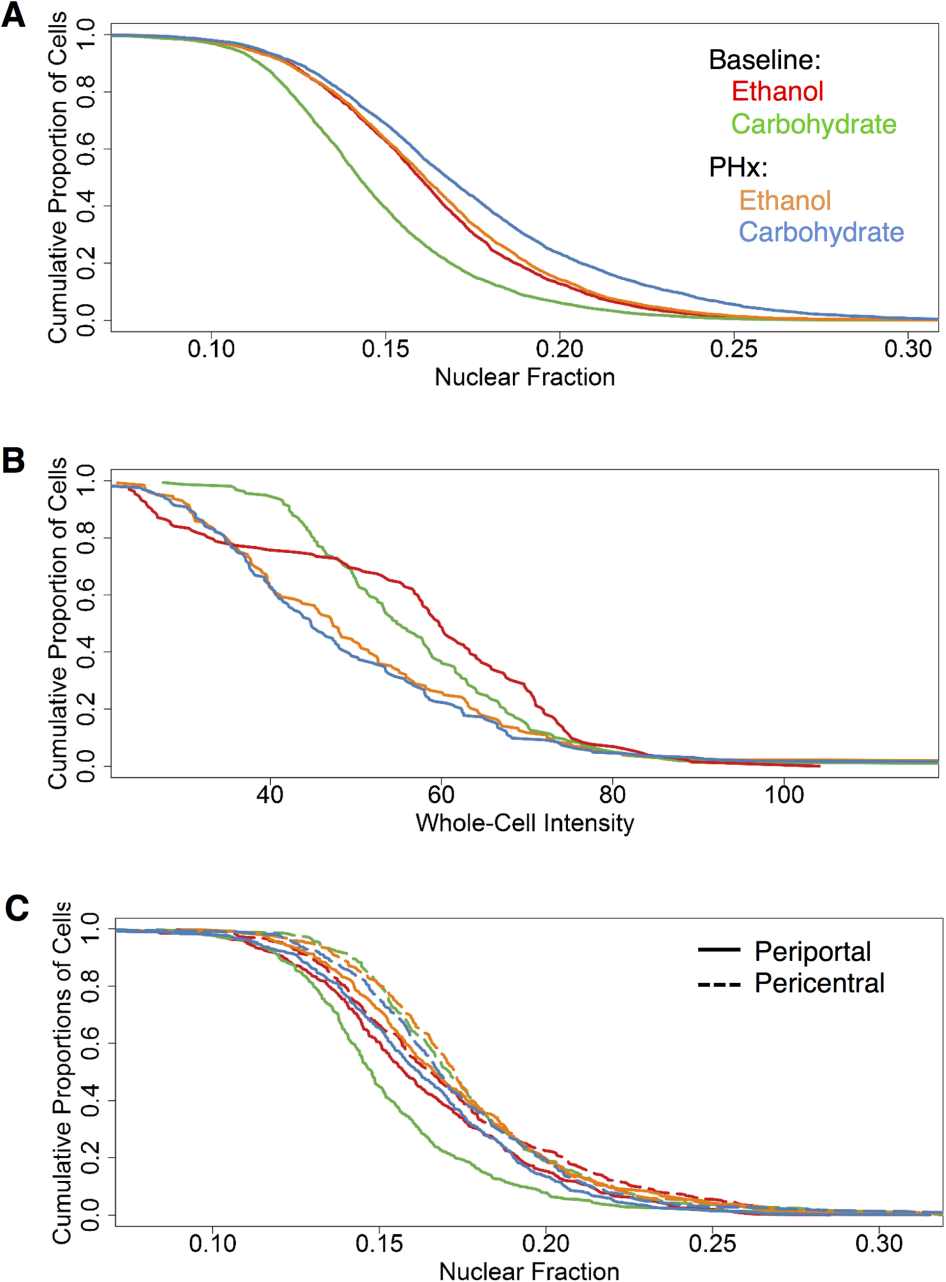
Nuclear fraction distribution of NF-κB in Hepatocytes and HSCs. **(A)** Nuclear fraction of intensities of Hepatocytes from both groups (red–baseline ethanol-adapted; green–baseline carbohydrate; orange–PHx ethanol-adapted; dark blue–PHx carbohydrate). Carbohydrate diet showed a higher level of nuclear fraction NF-κB than the ethanol group after 3h PHx. Scale bar: 40 μm. **(B)** Whole-cell intensities of HSCs from both baseline and post-PHx groups. Baseline adapted cells show the highest levels of NF-κB. **(C)** Nuclear fraction distribution of NF-κB in periportal (solid) and pericentral (dotted) cells of both baseline and post-PHx groups.

We collated the distributions of NF-κB nuclear fractions across all conditions for comparison (Fig 8). In the case of hepatocytes, the carbohydrate-fed animals showed the greatest difference in nuclear fraction between baseline and PHx, with the PHx group showing significantly higher nuclear fraction than the baseline (Fig 8A). In comparison, the ethanol-fed animals showed only a modest and non-significant difference in nuclear fraction between baseline and PHx. The carbohydrate PHx group showed the highest nuclear fraction overall (Fig 8A). In HSCs, PHx groups from both diet groups exhibited lower whole-cell NF-κB intensities compared to that of the baseline distribution (Fig 8B). In the ethanol-adapted condition, the liver showed higher NF-κB nuclear intensity compared to the pair-fed controls. However, by 3h post-PHx, adaptation to ethanol attenuated the NF-κB activity as shown by the relatively lower NF-κB nuclear localization compared to that of the carbohydrate group. We compared the nuclear fraction distributions of all the zonally segregated cell groups of the PHx and baseline conditions. At baseline, the ethanol adapted group showed a higher nuclear fraction of NF-κB compared to pair-fed control. In both diet groups, pericentral cells showed higher NF-κB localization (Fig 8C). We found that the PHx groups exhibited a marginally higher nuclear localization of NF-κB than that of the baseline groups. There was no marked distinction in zonal distribution of NF-κB localization between the diet groups post-PHx. However, pericentral cells in both diet groups showed higher nuclear localization in post-PHx as well.

#### Validation of NF-κB target gene expression

**Fig 9:**
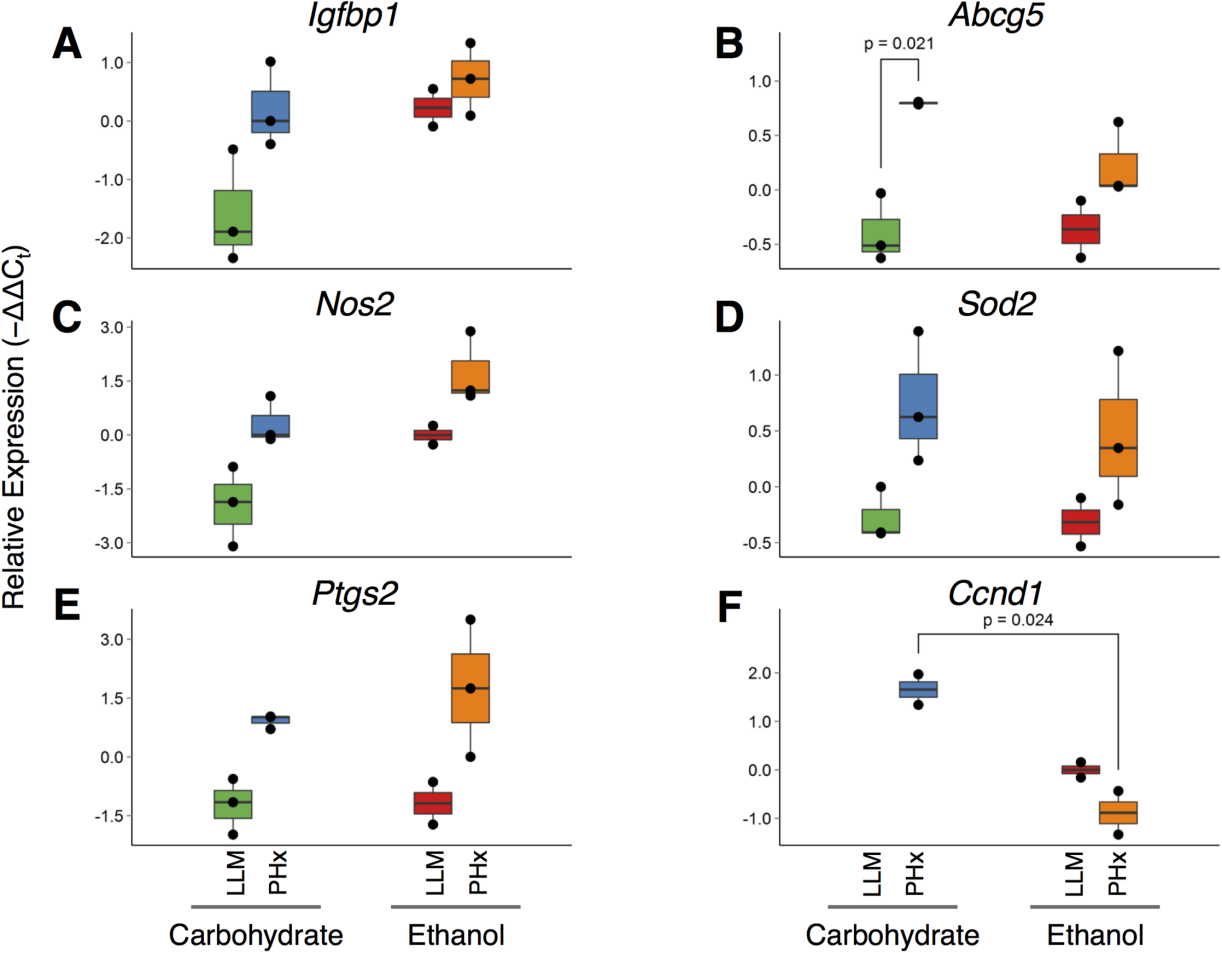
Validation of expression changes of putative NF-κB target genes in whole-tissue lysates by qPCR analysis. Results are presented as -ΔΔC_t_ values, where higher values correspond to increased expression, and lower values to decreased expression. Data were normalized by median-centering raw C_t_ values within each sample, followed by median-centering within each gene across all samples.

In order to test whether the changes observed in NF-κB are also reflected in its putative gene regulatory targets, we performed a high-throughput qPCR assay to measure the expression levels of several putative NF-κB target genes using whole tissue lysates. Multiple putative target genes displayed similar expression patterns consistent with changes in NF-κB, with increased expression in the post-PHx samples from both diets, and typically lower expression in the baseline samples; the baseline carbohydrate sample, in particular, routinely showed the lowest gene expression levels (Fig 9). It is important to note that the several putative target gene expression patterns differ from that of NF-κB activation distribution. This is likely due to a combinatorial action of multiple transcription factors involved in the activation of the selected genes, introducing another layer of complexity to their regulation [2]. In addition to these target genes, the expression patterns of select genes involved in liver regeneration and response to ethanol consumption were examined. Our analysis shows that *Ccnd1*, which promotes cell cycle progression, is undetectable in baseline carbohydrate samples but strongly upregulated following partial hepatectomy (Fig 9F). This increase is abrogated upon ethanol-adaptation, and decreases even further in post-PHx ethanol samples, consistent with decreased liver regeneration seen in alcoholic livers. By contrast, *Igfbp1*, an early response gene regulating IGF1 signaling, shows a dramatic increase post-PHx in both carbohydrate and ethanol-fed animals, as well as in the baseline ethanol samples (Fig 9A). These results are consistent with previously reported findings that demonstrate increased *Igfbp1* expression in humans following chronic ethanol consumption [48].

## Discussion

We investigated how the cell-cell variations in NF-κB activation within and across cell types are altered by adaptation to chronic ethanol intake. One of our main goals was to examine the zonal preference in NF-κB localization with ethanol intake followed by PHx. Such zonal differences in periportal and pericentral NF-κB levels can give more insight into the zonal bias in regulation at the transcriptional level of various enzymes activating metabolic and stress response pathways [18]. In addition to the heterogeneity of NF-κB localization in hepatocytes, we characterized the zonal variation in HSCs and examined whether NF-κB distribution showed a significant shift due to adaptation to chronic ethanol intake. Most of the studies associated with liver damage involve sample lysates, nuclear extracts and whole cell lysates [49–52]. The findings by those studies can be interpreted as a characteristic behavior of the tissue in its entirety. However, those results cannot account for the possibility that within a population of cells that constitute the tissue or cell aggregates, there may be multiple subpopulations, each exhibiting different levels of NF-κB activity. Varying levels of NF-κB activity may be an indicator of heterogeneous cellular function across the liver acinus. Our studies evaluated this possibility and identified the existence of subpopulations that vary in their NF-κB localization profiles as well as in yielding distinct responses across different cell types. The occurrence of cell sub-populations with varying intensities in the ethanol group indicated varying levels of NF-κB activation between cells as evidenced by the range of the intensity and nuclear fraction distributions.

The complex, organized structure of the liver gives rise to unique microenvironments where communities of heterogeneous cell populations carry out specific functions. The heterogeneity of hepatocyte distribution across zones is dynamic and could change depending on the pathological conditions [53]. Previous studies mainly focused on the zonal gene expression and the zonation of hepatic metabolism [18,19,21,22,54]. We were interested in investigating whether the expression changes were preceded by alterations in transcriptional activities due to zonal bias. To our knowledge, this is the first attempt in elucidating the potential zonal alterations of NF-κB localization in periportal and pericentral regions. We observed a preferential zonal localization towards the pericentral region in the basal state in both diet groups. This difference between zones was not as pronounced in the ethanol group as it was in the carbohydrate group. However, by comparison, the ethanol diet groups showed an overall higher pericentral NF-κB nuclear fraction than the carbohydrate control group in both adapted and in post-PHx groups. Previous results suggested that NF-κB may be activated predominantly in the periportal zones of the hepatic lobules at 1h post-PHx [29]. However, this zonal preference was not observed 3h post-PHx, even though an overall higher range of intensity was observed post-PHx compared to the baseline adapted state.

Previous single cell studies in mouse fibroblast cell lines demonstrated the heterogeneity in NF-κB activation and spontaneous oscillations arising due to the shuttling of NF-κB between cytoplasm and nucleus [55,56]. The heterogeneity of nuclear localization observed in our study could arise from variability of cellular responses distributed across such an oscillatory pattern. Further analysis using time series live cell imaging will be required to capture such a potential dynamic localization of NF-κB response in the liver. In the cell populations, the subpopulation with higher intensity is likely to contribute to the overall NF-κB activation levels leading to downstream response to PHx. The sub-population of cells with lower nuclear and cytosolic intensities in the ethanol group probably has little or no NF-κB activation.

We found that overall scaled intensity was higher in the ethanol-fed group compared to the pair-fed control group in hepatocytes. This was further supported by the occurrence of higher nuclear fractions in the ethanol-adapted group. The post-PHx results followed a similar pattern, with a subpopulation of cells in the ethanol-fed group exhibiting higher nuclear intensity distribution than other cells with the same cytoplasmic intensity. However, analysis of the nuclear fraction of NF-κB revealed that ethanol-fed animals exhibited an overlapping intensity distribution between PHx and baseline adapted states. Moreover, tissue from the pair-fed carbohydrate group showed a higher overall nuclear fraction, suggesting a reduction in the PHx-induced activation of NF-κB in the chronic ethanol-adapted animals. One likely explanation is that the chronic adaptation to ethanol intake mediated a faster decline in NF-κB localization post-PHx, resulting in an overall shift in the distribution towards lower levels than observed in the control group. It is also likely that the ethanol group may show lower NF-κB localization at earlier time points as well, exhibiting an even earlier deficiency in mounting a NF-κB response to PHx as demonstrated in previous experimental studies [13,17]. Experiments incorporating a time series design are required to resolve between these alternative scenarios.

Most of the previous studies on NF-κB activity in ethanol-adapted liver were conducted on whole tissue lysates rather than from single cell scale measures [50–52,57]. It has been widely demonstrated that variability at the single cell scale is much more extensive than what is typically observed across animals [23–25]. Therefore, the appropriate statistics involved in interpreting single cell data is not based on the number of animals, but considers the number of single cells. We also employed permutation analysis to overcome typical issues with incorrectly assuming that the variations arise due to a Normal/Gaussian distribution. In addition to hepatocytes, we extended our analysis to investigate NF-κB localization in HSCs. As in the case of hepatocytes, the majority of HSCs exhibited distinct sub-populations with varying intensity. The relative whole-cell intensity in the ethanol-adapted group was higher compared to that in the carbohydrate group. These findings support the conclusion that constitutive NF-κB levels increase within HSCs in response to chronic ethanol consumption. The lower whole-cell levels of NF-κB in HSCs at 3h post-PHx compared to baseline lead us to postulate that the contribution of HSCs to the inflammatory response post-PHx is minimal at this time point.

In summary, we used histopathological methods to obtain and analyze quantitative information on the localization of NF-κB p65 under different diet conditions across liver lobular locations and within two liver cell types. One of our primary aims was to investigate the zonal bias in NF-κB localization with ethanol intake in hepatocytes at baseline and post-PHx states. We found that the ethanol diet groups showed overall higher pericentral NF-κB nuclear fraction than in the carbohydrate control group in both adapted as well as post-PHx groups. We demonstrated that there are diverse levels of NF-κB localization post-PHx that show distinct activation in hepatocytes and HSCs, characterized by the intensity distributions of the cell subpopulations. Validation of these results by qPCR analysis in tissue lysates confirms that gene targets of NF-κB follow similar patterns of expression. Our cell type based analysis suggests that ethanol intake alone induces a higher rate of nuclear transport and activation of NF-κB, indicated by the robust shift in distribution of NF-κB localization in both hepatocytes and HSCs. However, this change was not sustained after 3h post-PHx indicating a deficiency in NF-κB localization and activation post-PHx in the ethanol group. The lower nuclear fraction intensity in the ethanol group suggested a faster decline in NF-κB activity around 3h post-PHx. Our findings demonstrated an alteration in the overall distribution of NF-κB localization as a result of adaptation to chronic ethanol intake. These zonal as well as cell type specific changes of NF-κB localization are likely to have distinct effects on the downstream targets mediating the observed deficiencies in response to PHx after chronic ethanol intake.

## Supporting Information

S1 Fig NF-κB localization in baseline carbohydrate and ethanol-adapted rat liver tissue, at the baseline and 3h after PHx. Average nuclear fraction data corresponding to the distributions shown in Fig 2C and 2D, and Fig 5C and 5D. Error bars represent 95% confidence intervals.
(TIF)

S1 Table Primer designs used for qPCR analysis of putative NF-κB target genes. (PDF)

## Abbreviations

ALD: alcoholic liver disease
IKB: inhibitor of kappa B
IKK: inhibitor of kappa B kinase
HIF-*α*: hypoxia inducible factor- alpha
HSC: hepatic stellate cells
LLM: left lateral and medial lobes
NF-κB: nuclear factor kappa B
PHx: partial hepatectomy
STAT3: Signal transducer and activator of transcription 3
TNF-α: tumor necrosis factor–alpha
