# Supplementary figures and images for: In Vivo Zonal Variation and Liver Cell-Type Specific NF-κB Localization after Chronic Adaptation to Ethanol and following Partial Hepatectomy
Source: PLoS One. 2015 Oct 9;10(10):e0140236. doi: 10.1371/journal.pone.0140236 (PMC4599916; doi:10.1371/journal.pone.0140236)

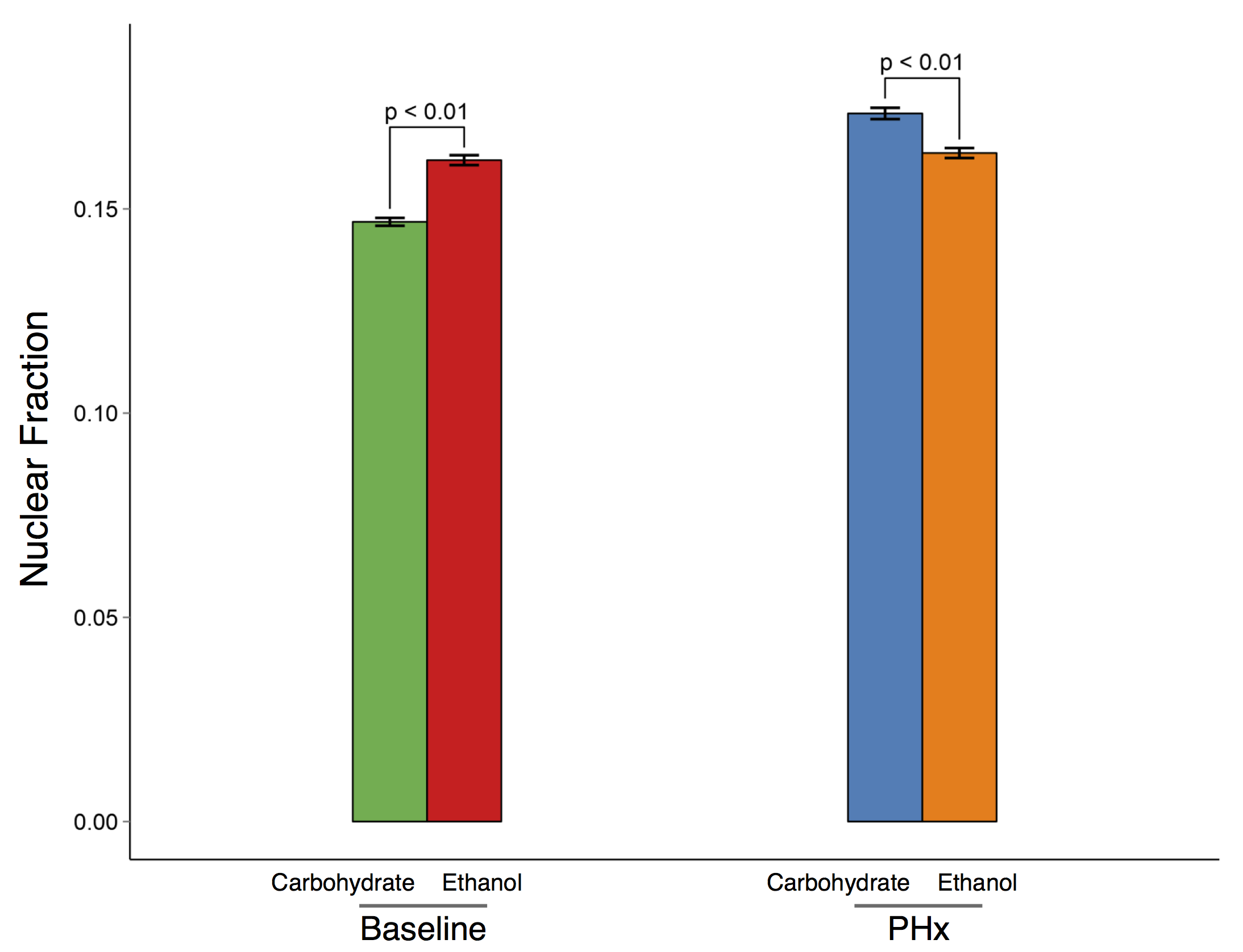

Supplement: S1 Fig — Average nuclear fraction data corresponding to the distributions shown in Fig 2C and 2D, and Fig 5C and 5D. Error bars represent 95% confidence intervals. (TIF) [file pone.0140236.s001.tif]
