# Supplementary material for: In Vivo Zonal Variation and Liver Cell-Type Specific NF-κB Localization after Chronic Adaptation to Ethanol and following Partial Hepatectomy
Source: PLoS One. 2015 Oct 9;10(10):e0140236. doi: 10.1371/journal.pone.0140236 (PMC4599916; doi:10.1371/journal.pone.0140236)
Supplement: S1 Table — (PDF) [file pone.0140236.s002.pdf]

Table S1. Primer designs used for qPCR analysis of putative NF-κB target genes.

**Gene**	**Forward Sequence**	**Reverse Sequence**
Igfbp1	5'-CTGCCGCTCAACAGAAAGCA-3'	5'-CTCCATGGGTAGACACACCAG-3'
Abcg5	5'-GGGAAGTGTTTGTGAACGGC-3'	5'-GTGTATCTCAGCGTCTCCCG-3'
Nos2	5'-GAGTTCCCATCATTGCGTGT-3'	5'-AGTAGTAGCGGGGCTTCAGA-3'
Sod2	5'-GCATTTTCTGGACAAACCTGA-3'	5'-CTCCAGCAACTCTCCTTTGG-3'
Ptgs2	5'-GTTCATCCCGGATCCCCAAG-3'	5'-AGGATACACCTCTCCACCGA-3'
Ccnd1	5'-GCGCCCTCCGTTTCTTACTT-3'	5'-TCGCAGACCTCTAGCATCCA-3'
